# Breast cancer among former college athletes compared to non-athletes: a 15-year follow-up

**DOI:** 10.1054/bjoc.1999.0987

**Published:** 2000-01-18

**Authors:** G Wyshak, R E Frisch

**Affiliations:** Department of Population and International Health and Center for Population and Development Studies, Harvard School of Public Health, 665 Huntingdon Avenue, Boston, MA, 02115, USA; Department of Psychiatry, Harvard Medical School, Boston, MA, USA

**Keywords:** breast cancer, former college athletes

## Abstract

A growing body of evidence indicates that physical activity is protective against breast cancer. In 1996–97, we conducted a 15-year follow-up of 5398 college alumnae comprised of former college athletes with their non-athletic classmates. Participants completed a detailed mailed questionnaire on their health history from 1981–82 to the present. Excluding women who had died and non-deliverable questionnaires, 84.7% (*n* = 3940) of the participants in our earlier study responded to the questionnaire; the response rate for former athletes was 86.6% (*n* = 1945), for non-athletes, 83.0% (*n* = 1995). Results confirmed our earlier findings. Based on self-reports, former college athletes had a significantly lower risk of breast cancer than the non-athletes. The OR for the 15-year incidence of breast cancer is 0.605 with 95% confidence interval (CI) (0.438–0.835); the 15-year incident breast cancers were 64 among the athletes and 111 among the non-athletes. Among women under 45 the protective effect of physical activity on the risk of breast cancer is considerably greater; odds ratio (OR) = 0.164, 95% CI (0.042–0.636). Athletic activity during the college and pre-college years is protective against breast cancer throughout the life span, and more markedly among women under 45. These results confirm our earlier findings and the findings of other investigators. © 2000 Cancer Research Campaign


*British Journal of Cancer* (2000) **82**(3), 726–730
© 2000 Cancer Research Campaign
DOI: 10.1054/ bjoc.1999.0987, available online at http://www.idealibrary.com on IDEAL®

### Breast cancer among former college athletes compared to non-athletes: a 15-year follow-up

**G Wyshak[1,3] and RE Frisch[2]**

[1]Department of Population and International Health and [2]Center for Population and Development Studies, Harvard School of Public Health, 665 Huntingdon Avenue, Boston, MA 02115, USA; [3]Department of Psychiatry, Harvard Medical School, Boston, MA, USA

**Summary** A growing body of evidence indicates that physical activity is protective against breast cancer. In 1996–97, we conducted a 15-year follow-up of 5398 college alumnae comprised of former college athletes with their non-athletic classmates. Participants completed a detailed mailed questionnaire on their health history from 1981–82 to the present. Excluding women who had died and non-deliverable questionnaires, 84.7% ($n$ = 3940) of the participants in our earlier study responded to the questionnaire; the response rate for former athletes was 86.6% ($n$ = 1945), for non-athletes, 83.0% ($n$ = 1995). Results confirmed our earlier findings. Based on self-reports, former college athletes had a significantly lower risk of breast cancer than the non-athletes. The OR for the 15-year incidence of breast cancer is 0.605 with 95% confidence interval (CI) (0.438–0.835); the 15-year incident breast cancers were 64 among the athletes and 111 among the non-athletes. Among women under 45 the protective effect of physical activity on the risk of breast cancer is considerably greater; odds ratio (OR) = 0.164, 95% CI (0.042–0.636). Athletic activity during the college and pre-college years is protective against breast cancer throughout the life span, and more markedly among women under 45. These results confirm our earlier findings and the findings of other investigators. © 2000 Cancer Research Campaign

**Keywords:** breast cancer; former college athletes

A relation between physical activity and risk of breast cancer in women was first reported in 1985 when Frisch et al (1985) found that former college athletes had a significantly lower risk of breast cancer than non-athletes. The study was suggested by previous findings that strenuous exercise delays menarche (Frisch et al, 1980; Brinton, 1994) and that physical activity may affect oestrogen metabolism (Frisch et al, 1981). Experimental and epidemiologic data have suggested that the effect of physical activity on breast cancer could be mediated through changes in endogenous hormones related to body composition, particularly a reduced level of body fat, and immunologic parameters (Friedenreich et al, 1998). Limited evidence of improved immune function in athletes indicates that macrophages, natural killer cells, lymphokine-activated cells and their regulating cytokines, neutrophils and acute-phase proteins increase in number and/or activity in response to exercise (Shephard and Shek, 1995).

In a review evaluating and updating the epidemiologic studies on recreational and occupational physical activities and breast cancer development Gammon et al (1998) report a more than twofold increase in the number of such studies since the reviews published in the years 1995–1997. These authors conclude that 'most epidemiologic studies of physical activity reported a reduction in the risk of breast cancer among physically active women.' A recent review of the epidemiologic results on physical activity and breast cancer reported that 15 of 21 studies suggested that physical activity reduces the risk of breast cancer (Friedenrich et al, 1998); this review included many of the studies also evaluated by Gammon et al (1998).

We now report data on a 15-year follow-up of the original 5398 participants of our earlier study (Frisch et al, 1985), who were then 21–80 years of age. This study, covering the participants' health history from 1981–82 to the present, investigated the relation between physical activity and breast cancer among former college athletes and non-athletes 15 years after our first inquiries. The data are based on self-reports.

### SUBJECTS AND METHODS

Our study conducted in 1981–82 (Frisch et al, 1985) comprised a detailed questionnaire sent in December 1981 to 7359 alumnae listed as currently alive by the alumnae office of eight colleges and two universities. Of the 5398 respondents 2622 were former athletes and 2776 were former non-athletes who resided in the USA and abroad. The response rate was 71.4%; 71.9% for the athletes and 70.1% for the controls.

A follow-up questionnaire in 1996 queried the 5398 participants in our earlier study about their health history from 1981–82 to the present. The questionnaire included inquiry about the following: occupation and living arrangements; current exercise or athletic activity; medical history; weight gain or loss; family history of cancer, cardiovascular disease, diabetes and other medical conditions; medications taken; diet including alcohol intake; reproductive history; menstruation and menopause; oestrogen therapy for the menopause; pregnancy history; contraception and fertility/infertility history; activities (walking, jogging, running, etc); physical limitations; mental health; self-assessment of general health.

The current addresses of athletes and non-athletes were obtained from each of the ten participating colleges and universities. In

*Received 6 April 1999*
*Revised 20 July 1999*
*Accepted 22 July 1999*

*Correspondence to:* G Wyshak

**Table 1** Characteristics of athletes and non-athletes

| | Athletes (*n*=1945) | | Non-Athletes (*n*=1995) | | | |
|---|---|---|---|---|---|---|
| | **Mean** | **SE** | **Mean** | **SE** | ***P*-value** | **95% CL** |
| Age at time of reporting | 52.51 | 0.28 | 54.67 | 0.27 | 0.0001 | (–2.92,–1.40) |
| Year of birth | 1943.76 | 0.28 | 1941.6 | 0.27 | 0.0001 | (1.40,2.92) |
| *Age adjusted* | | | | | | |
| | % | | % | | | |
| Cancer in family | 46.98 | | 47.78 | | 0.6142 | |
| Cancer, mother | 26.28 | | 26.56 | | 0.842 | |
| Breast cancer, mother | 12.58 | | 12.29 | | 0.7817 | |
| Breast cancer, sister | 3.17 | | 3.78 | | 0.2907 | |
| Breast cancer, family | 15.9 | | 15.82 | | 0.9434 | |
| | Mean | SE | Mean | SE | | 95% CL |
| Age at menarche (years) | 13.14 | 0.030 | 12.93 | 0.030 | 0.0001 | (0.13,0.29) |
| | % | | % | | | |
| Used contraception | 22.37 | | 22.6 | | 0.8497 | |
| <60, contraception | 23.45 | | 32.63 | | 0.5617 | |
| HRT | 29.87 | | 33.63 | | 0.0073 | |
| 50+, HRT | 51.12 | | 50.22 | | 0.6734 | |
| Ever-smoked | 41.27 | | 42.6 | | 0.3808 | |
| Current exercise | 73.51 | | 63.27 | | 0.0001 | |
| Dietary restriction | 46.05 | | 51.70 | | 0.0004 | |
| Low fat diet | 37.19 | | 39.88 | | 0.0798 | |
| Drink alcohol | 79.09 | | 77.78 | | 0.3204 | |
| | Mean | SE | Mean | SE | | 95% CL |
| Height, cm | 166.84 | 0.144 | 165.35 | 0.143 | 0.0001 | (1.09,1.89) |
| Weight, kg | 64.75 | 0.266 | 63.79 | 0.263 | 0.01 | (0.23,1.69) |
| Per cent fat | 37.53 | 0.124 | 38.16 | 0.123 | 0.0003 | (–0.97,–0.29) |
| BMI | 23.25 | 0.091 | 23.32 | 0.090 | 0.5678 | (–0.32, 0.18) |
| Per cent ever-pregnant | 79.6 | | 81.39 | | 0.1521 | |
| If ever-pregnant | | | | | | |
| | Mean | SE | Mean | SE | | 95% CL |
| No. pregnancies | 3.12 | 0.039 | 2.98 | 0.037 | 0.0134 | (0.03,0.25) |
| No. live births | 2.56 | 0.028 | 2.41 | 0.027 | 0.0001 | (0.07, 0.23) |
| Age first pregnancy | 28.03 | 0.121 | 28.17 | 0.118 | 0.4308 | (–0.47,0.19) |
| *Over 50 years of age* | | | | | | |
| | % | | % | | | |
| Any menopause* | 94.23 | | 91.53 | | 0.025 | |
| Natural menopause | 94.12 | | 91.34 | | 0.0263 | |

*Includes surgical menopause.

addition, the colleges informed us of women who had died between 1982 and 1995, with dates of death when available. Data collection commenced in October 1996 and continued through April 1997. Three questionnaires were received after the closing date, and are not included in the analysis. A total of 268 women had died (5.0%): 121 athletes and 147 non-athletes. In addition, 480 women (8.9%) had non-deliverable and/or no follow-up addresses from the colleges.

Of the 4650 living alumnae who received questionnaires, 3940 responded with completed questionnaires (12 women returned blank questionnaires). The questionnaire was re-sent to those women not responding to our first request. Thus, for the 15-year follow-up, the response rate was 3940/4650 (84.7%). The response rates for the former college athletes and the non-athletes were respectively 86.6% ($n$ = 1945) and 83.0% ($n$ = 1995), a difference which is not statistically significant.

All data for both the 1982 study and the current study were kept confidential. However, using identification numbers, we were able to link the two data sets.

We report the findings on breast cancer among the 3940 respondents in this 15-year follow-up study.

### Statistical methods

Fifteen-year incidence rates, based on the follow-up of the subjects, are computed for athletes and non-athletes in five age groups: (1) under 45 years, (2) 45–49, (3) 50–54, (4) 55–64, (5) 65 and older. The rates are the number of cases divided by the number of women in the age group. Odds ratios (OR) for comparing the athletes and non-athletes are coded, with athletes given the value 1, non-athletes the value 0. Odds ratios controlling for age only were computed, and multiple logistic regression analyses were carried out to determine the effects of possible confounding factors on the risk of breast cancer. These factors included the following: age in single years, ever-pregnant, family history of breast cancer, ever-smoked, current exercise; for women under 45: ever-used contraceptives; for women 45 and older: ever-used hormonal replacement therapy (HRT) for the menopausal and post-menopausal periods, menopausal status, per cent body fat (estimated by the equations of Cohn et al, 1980 and Ellis, 1974). Except for age and per cent body fat variables were dichotomized – 1/0 – according to presence or absence of the factor. Statistical methods included simple descriptive statistics, analysis of covariance and multivariate logistic regression. The analyses were carried out on a PC using SAS software (1987).

*© 2000 Cancer Research Campaign*

**Table 2** Age-specific 15-year incidence rates of breast cancer of former college athletes and non-athletes

| Age at time of reporting years | Athletes (*n*=1935)* | | Non-Athletes (*n*=1973)* | |
|---|---|---|---|---|
| | Number | Rate/1000 | Number | Rate/1000 |
| <45 | 3 | 4.3 | 9 | 23.3 |
| 45–49 | 5 | 17.4 | 8 | 24.1 |
| 50–54 | 11 | 47.2 | 27 | 55.8 |
| 55–64 | 21 | 65.8 | 30 | 81.5 |
| ≥65 | 24 | 60.3 | 37 | 91.8 |
| Total | 64 | 32.9 | 111 | 56.3 |

*Excludes the 32 women (ten athletes and 22 non-athletes) who reported breast cancer in the earlier study.

### RESULTS

The characteristics of the participants (Table 1) are adjusted for age in single years by analysis of covariance. The former college athletes are slightly younger than the non-athletes. This age difference is accounted for by the inclusion of alumnae of a physical training college, most of whom were athletes, and most of whom tended to be younger than other participants.

The former college athletes and their non-athlete classmates are similar with respect to family history of cancer and breast cancer, ever-pregnant, use of contraceptives, and the use of HRT by women 50 and older. With respect to life-style factors such as exercise, smoking and diet, the proportion of former athletes who currently exercise was higher than that of non-athletes. The two groups did not differ on smoking history, that is, whether they ever smoked. More non-athletes than athletes reported dietary restrictions and being on a low fat diet.

The former athletes are taller and heavier than the non-athletes, but are leaner as assessed by the equations of Cohn et al (1980) and Ellis et al (1974) (see Appendix for Cohn and Ellis's equations), but not by body mass index (BMI). Among women 50 years of age and older, HRT use did not differ. Although the proportions of ever-pregnant women do not differ significantly, former athletes had slightly more pregnancies and slightly more live births. Age at first pregnancy is similar in the two groups.

Age at first pregnancy showed no relation to breast cancer among women who had at least one pregnancy (80% of the entire sample), controlling for age and for status as former athlete or non-athlete. Among those who reported breast cancer the mean (and standard error) for age at first pregnancy ($27.69 \pm 0.36$) was similar to those without breast cancer ($28.13 \pm 0.09$).

Table 2 shows the frequencies and rates per 1000 for the 15-year incident non-fatal breast cancers, that is, breast cancer among survivors of breast cancer who responded to the self-administered questionnaire. The 15-year incidence rates exclude the 32 women who reported breast cancer in the earlier study and responded to the follow-up. No women under 45 reported breast cancer in the earlier study (Frisch et al, 1985).

Table 3 presents the findings of the relation between physical activity and breast cancer, adjusting only for age. Former college athletes are at a significantly lower risk of breast cancer than their non-athletic counterparts, for the 15-year incidence (denoted breast cancer in 1982 and after, in Table 3). The OR, adjusting for age only, is 0.606 (95% CI 0.442–0.832, $P = 0.0019$). Among women who are now under 45 years of age (born 1952 or after), the OR for breast cancer is most striking – the age-adjusted OR is 0.194 (95% CI 0.052–0.723, $P = 0.0145$).

Table 3 presents multivariate OR determined by logistic regression, controlling for possible confounding factors. The following variables were included in the logistic model in addition to athlete/non-athletes: age in single years, ever-pregnant, ever-smoked, current exercise, menopausal status, use of contraceptives, use of HRT, per cent body fat (in single units) and family history of breast cancer.

Among women of all ages, for the 15-year incidence of breast cancers, i.e. breast cancer which occurred in 1982 and after, OR = 0.605 (95% CI 0.438–0.835, $P = 0.0023$). For women under 45, the multivariate OR is 0.164 (95% CI 0.042–0.636, $P = 0.0089$). Of the women under 45 (born 1952 or after) none had reported breast cancer in the 1981–82 study. That is, among women under 45 years of age, the non-athletes' risk of breast cancer is more than 6 times that of the former college athletes, adjusting for possible confounders.

**Table 3** Odds ratios for breast cancer diagnosed after 1981 in athletes/non-athletes

| | All Women | | | | Ever-Pregnant Women | | | |
|---|---|---|---|---|---|---|---|---|
| | Odds ratio | 95% Confidence limits | | *P*-value | Odds ratio | 95% Confidence limits | | *P*-value |
| | | Lower | Upper | | | Lower | Upper | |
| Age-adjusted in single years | | | | | | | | |
| All Ages | 0.606 | 0.442 | 0.832 | 0.0019 | 0.571 | 0.405 | 0.805 | 0.0014 |
| Ages Under 45 | 0.194 | 0.052 | 0.723 | 0.0145 | 0.466 | 0.103 | 2.118 | 0.323 |
| Multivariate odds ratios* | | | | | | | | |
| All Ages | 0.605 | 0.438 | 0.835 | 0.0023 | 0.575 | 0.405 | 0.815 | 0.0019 |
| Ages Under 45 | 0.164 | 0.042 | 0.636 | 0.0089 | 0.466 | 0.097 | 2.232 | 0.339 |
| Multivariate odds ratios excluding per cent body fat | | | | | | | | |
| All Ages | 0.601 | 0.436 | 0.827 | 0.0018 | 0.561 | 0.397 | 0.794 | 0.0011 |
| Ages Under 45 | 0.161 | 0.042 | 0.618 | 0.0078 | 0.419 | 0.088 | 1.989 | 0.2739 |

N.B. Age refers to age at time of reporting. *Adjusted for age in single years, ever-pregnant, use of contraceptives, use of HRT, family history of breast cancer, current exercise, ever smoked, per cent body fat. For those 45 and over excludes use of contraceptives; for those under 45 excludes use of HRT.

© 2000 Cancer Research Campaign

Table 3 shows the results excluding per cent fat as a covariate. This was done because there were missing data on weight and height for 102 women and therefore it was not possible to compute per cent fat. The inclusion of covariates changed the OR relatively little, as may be noted by comparing the results in Table 3.

Table 3 also presents the results (and ORs) for women who had ever been pregnant, 81% of the entire group of 3940 women. The OR for the 15-year incidence is 0.571; the OR for ever-pregnant women differed negligibly from the OR for all women (shown in Table 3). Among women under 45 where the OR = 0.466, the 95% CI of 0.103–2.118, $P = 0.323$ shows the protective effect of being a former athlete, but is not statistically significant due to small numbers among ever-pregnant women under 45.

Current exercise (reported as doing regular exercise at time of response to the questionnaire), adjusting for age and in a multivariate model not including athlete/non-athlete as a covariate, was not significantly associated with breast cancer.

### DISCUSSION

To our knowledge we were the first investigators to examine the possible relation between physical activity and breast cancer, using data from a retrospective cohort study. Our 1985 paper, (Frisch et al), reported that women who had participated in organized athletic activity while in college (82% of whom had begun their athletic training in high school or earlier) had a significantly lower lifetime occurrence of cancers of the breast than their non-athletic classmates. Our follow-up of these subjects carried out 15 years later confirms the previously reported finding that the risk of breast cancer is significantly lower among former college athletes than among non-athletes.

In the past 15 years, 15 of 21 epidemiologic studies have shown a protective effect of recreational and occupational physical activities on the risk of breast cancer (Friedenreich et al, 1998; Gammon et al, 1998). As Gammon et al (1998) pointed out, differences in outcome could be due to the methods used in the assessment of physical activity, whether the activity was recreational or occupational, the optimal time period, duration, frequency or intensity and the time period in a woman's life during which she was exposed to a risk factor or protective factor. In contrast to the majority of studies (Friedenreich et al, 1998; Gammon et al, 1998), Rockhill et al (1998) did not support a link between physical activity and breast cancer. These authors analysed data from the Nurses' Health Study II, a prospective study of women aged 25–42 in 1989, who were followed for 6 years. Physical activity during high school and at ages 18–22 was assessed by asking: 'How often did you participate in strenuous physical activity at least twice a week?' These authors conclude that their findings do not support a link between physical activity in late adolescence (defined as ages 18–22), or in the recent past, and breast cancer risk among young adult women.

Our studies avoid many of the pitfalls cited by Gammon et al (1998), and Friedenreich et al (1998). The two major comparison groups, women who were former college athletes and women who were non-athletic, met rigorously defined criteria for physical activity in college and high school or earlier. The former athletes were identified from college athletic department records; and we verified the assignment to athlete/non-athlete status by examining women's responses to our questionnaire; thus, our procedures minimized recall bias and the risk of misclassification. Our study design is a retrospective cohort. In this 15-year follow-up the response rate was 84.4%. The detailed questionnaires that we used in both studies provide data that permit us to control for possible confounding factors. The two groups of women are similar with respect to social and economic characteristics. The results reported here, which confirm our earlier findings (Frisch et al, 1985), have biological plausibility – the former athletes were leaner at all ages, had later menarche and earlier menopause, all of which are factors associated with a lower risk of breast cancer (Frisch et al, 1985).

A new finding from this study concerns the importance of the timing of physical activity. Contrary to Thune et al (1997), we found that current regular exercise was not protective against breast cancer after adjusting for whether or not a woman was a former college athlete or a non-athlete. Differences in the timing of physical activity, as discussed above, may contribute to the conflicting findings on the relation between physical activity and breast cancer (Friedenreich et al, 1998, Gammon et al, 1998).

Related to the role of timing between physical activity and the occurrence of breast cancer is our present finding that the greatest difference in risk between former athletes and non-athletes occurs at age under 45. This result is in accord with Bernstein et al (1994) who reported an overall relative risk of 0.42 for active women under 40, and Thune et al (1997) who found the reduction in risk was greater in younger women than older women.

Obesity is a breast cancer risk factor (Frisch et al, 1985; Barash-Ballard and Swanson, 1996; Hershcopf and Bradlow, 1987). Our athletes and non-athletes did not differ significantly in BMI (weight in kilograms divided by height in metres squared). However, muscles are heavy (80% water, compared to 5–10% fat) and the BMI of an athlete can be misleading as to per cent fat. A study of young athletes, compared to controls, using MRI (magnetic resonance imaging) for direct measurements of fat showed athletes had 30–40% less fat at the same weight as controls (Frisch et al, 1993). When we used the equations of Cohn et al (1980) and Ellis et al (1974), to determine per cent body fat, we found that former athletes had significantly less body fat. (To estimate per cent body fat using the equations of Cohn et al, the measurements needed are weight, height and age.) The degree of fatness has been related to the risk of breast cancer.

In summary, women who are former college athletes have a significantly lower risk of breast cancer, as compared with women who were non-athletes, throughout the age span.

Further research on the time-dependency between physical activity and breast cancer is needed. We estimate from our findings that the so-called 'Prevented Fraction' (Kleinbaum et al, 1982), the proportion of breast cancers that would be prevented if women were exposed to physical activity in the college years or earlier would be 17%; for women under 45 years of age about 50%. Based on these estimates of the 'Prevented Fraction', the importance of regular, moderate physical activity initiated in the college and pre-college years may have lasting impacts throughout the life span of women. The confirmation of our earlier results (Frisch et al, 1985) can be taken as strong evidence for the relationship between athletic activity started in adolescence. This evidence has implications for public health and health promotion and should be used as a basis for policy.

© 2000 Cancer Research Campaign 

### REFERENCES

Ballard-Barbash R and Swanson SA (1996) Body weight: estimation of risk for breast and endometrial cancers. *Am J Clin Nutr* **63**: 437S–441S

Bernstein L, Henderson BE, Hanisch R, Sullivan-Halley J and Ross RK (1994) Physical exercise and reduced risk of breast cancer in young women. *J Natl Cancer Inst* **86**: 1403–1408

Brinton LA (1994) Ways that women may possibly reduce their risk of breast cancer. *J Natl Cancer Inst* **86**: 1371–1372

Cohn SH, Vartsky S, Yasumura A et al (1980) Compartmental body composition based on total-body nitrogen, potassium and calcium. *Am J Physiol* **239**: E524–530

Ellis KJ, Shukla KK, Cohn SH and Pierson RN Jr (1974) A predictor for total body potassium in man based on height, weight, sex and age: applications in metabolic disorders. *Lab Clin Med* **83**: 716–727

Friedenreich CM, Thune I, Brinton LA and Albanes D (1998) Epidemiologic issues related to the association between physical activity and breast cancer. *Cancer* (suppl) **83**: 600–610

Frisch RE, Gotz-Welbergen AV, McArthur JW, Albright T, Witschi J, Bullen B, et al (1981) Delayed menarche and amenorrhea of college athletes in relation to age at onset of training. *JAMA* **246**: 1559–1563

Frisch RE, Snow RC, Johnson LA, Gerard B, Barbieri R and Rosen B (1993) Magnetic resonance imaging of overall and regional body fat, estrogen metabolism, and ovulation of athletes compared to controls. *J Clin Endocrin Metab* **77**: 471–477

Frisch RE, Wyshak G, Albright NL, Albright TE, Schiff I, Jones KP, et al (1985) Lower prevalence of breast cancer and cancers of the reproductive system among former college athletes compared to non-athletes. *Br J Cancer* **52**: 885–891

Frisch RE, Wyshak G and Vincent L (1980) Delayed menarche and amenorrhea in ballet dancers. *N Engl J Med* **303**: 17–19

Gammon MD, John EM and Britton JA (1998) Recreational and occupational physical activities and risk of breast cancer. *J Natl Cancer Inst* **90**: 100–117

Hershcopf RJ and Bradlow HL (1987) Obesity, diet, endogenous estrogens and the risk of hormone sensitive cancer. *Am J Clin Nutr* **45**: 283–289

Kleinbaum DG, Kupper LL and Morgenstern H (1982) *Epidemiologic Research: Principles and Quantitative Methods* pp., 164–166. Lifetime Learning: Belmont, California

Rockhill B, Willett WC, Hunter DJ et al (1998) Physical activity and breast cancer risk in young adult women. *J Natl Cancer Inst* **90**: 1155–1160

SAS (1987) Statistical Analysis System: Cary, NC

Shephard RJ and Shek PN (1995) Cancer, immune function and physical activity. *Can J Applied Physiol* **20**: 1–25

Thune I, Brenn T, Lund E and Gaard M (1997) Physical activity and the risk of breast cancer. *N Engl J Med* **336**: 1269–1275

### ACKNOWLEDGEMENTS

We thank the Alumnae Offices of the following Colleges: Barnard, Bryn Mawr, Mount Holyoke, Radcliffe, Smith, Vassar, Wellesley and Springfield; and the Universities of Southern California and Wisconsin for their generous cooperation in providing us with up-to-date addresses for living alumnae and information about alumnae who are deceased. We especially thank Professor Frederick Mosteller for his interest and support throughout this project and for his very helpful remarks on the manuscript. We also thank Drs Robert Barbieri, Mark Hornstein, Graham Colditz, and Nile Albright for helpful comments on the questionnaire. We appreciate the assistance of Marie McPherson and Kelly Blanchard, Project Managers in the data collection phase of the research, and the clerical assistance of LaGhistla Williams. This research was supported in part by the Advanced Medical Research Foundation, Boston, MA and in part by the Harvard School of Public Health.

### APPENDIX

Predictions of body composition by equations of Cohn et al (1980) and Ellis et al (1974).

Predicted potassium, $K_p = aW^{1/2}\,Ht^2$;

W = weight (kg)

Ht = height (metres)

a (for females) = 4.58 − 0.010 Age (y).

Lean body mass (LBM) (for females) = $K_p \times 0.442$ kg.

Fat (kg) = body weight (kg) – LBM (kg).

%Fat = Fat/body weight.

*© 2000 Cancer Research Campaign*
